# Inhibition of *Aspergillus flavus* Growth and Aflatoxin Production in *Zea mays* L. Using Endophytic *Aspergillus fumigatus*

**DOI:** 10.3390/jof8050482

**Published:** 2022-05-06

**Authors:** Amer M. Abdelaziz, Deiaa A. El-Wakil, Mohamed S. Attia, Omar M. Ali, Hamada AbdElgawad, Amr H. Hashem

**Affiliations:** 1 Botany and Microbiology Department, Faculty of Science, Al-Azhar University, Cairo 11884, Egypt; amermorsy@azhar.edu.eg; 2 Department of Biology, Faculty of Science, Jazan University, Jazan 82817, Saudi Arabia; de107@yahoo.com; 3 Plant Pathology Research Institute, Agricultural Research Center, Giza 12619, Egypt; 4 Department of Chemistry, Turabah University College, Turabah Branch, Taif University, P.O. Box 11099, Taif 21944, Saudi Arabia; 5 Integrated Molecular Plant Physiology Research (IMPRES), Department of Biology, University of Antwerp, 2000 Antwerp, Belgium; hamada.abdelgawad@uantwerpen.be; 6 Department of Botany, Faculty of Science, Beni-Suef University, Salah Salem St., Beni-Suef 62511, Egypt

**Keywords:** maize, fungal infection, ultra-structural features, aflatoxins, *Aspergillus fumigatus*

## Abstract

*Aspergillus flavus* infection of vegetative tissues can affect the development and integrity of the plant and poses dangerous risks on human and animal health. Thus, safe and easily applied approaches are employed to inhibit *A*. *flavus growth.* To this end, the fungal endophyte, i.e., *Aspergillus fumigatus,* was used as a safe biocontrol agent to reduce the growth of *A. flavus* and its infection in maize seedlings. Interestingly, the safe endophytic *A. fumigatus* exhibited antifungal activity (e.g., 77% of growth inhibition) against *A. flavus.* It also reduced the creation of aflatoxins, particularly aflatoxin B_1_ (AFB_1_, 90.9%). At plant level, maize seedling growth, leaves and root anatomy and the changes in redox status were estimated. Infected seeds treated with *A. fumigatus* significantly improved the germination rate by 88.53%. The ultrastructure of the infected leaves showed severe disturbances in the internal structures, such as lack of differentiation in cells, cracking, and lysis in the cell wall and destruction in the nucleus semi-lysis of chloroplasts. Ultrastructure observations indicated that *A. fumigatus* treatment increased maize (leaf and root) cell wall thickness that consequentially reduced the invasion of the pathogenic *A. flavus*. It was also interesting that the infected seedlings recovered after being treated with *A. fumigatus*, as it was observed in growth characteristics and photosynthetic pigments. Moreover, infected maize plants showed increased oxidative stress (lipid peroxidation and H_2_O_2_), which was significantly mitigated by *A. fumigatus* treatment. This mitigation was at least partially explained by inducing the antioxidant defense system, i.e., increased phenols and proline levels (23.3 and 31.17%, respectively) and POD, PPO, SOD and CAT enzymes activity (29.50, 57.58, 32.14 and 29.52%, respectively). Overall, our study suggests that endophytic *A. fumigatus* treatment could be commercially used for the safe control of aflatoxins production and for inducing biotic stress tolerance of *A. flavus*-infected maize plants.

## 1. Introduction

Phytopathogenic fungi negatively affect plant growth and yield. *A. flavus*, *Penicillium*, and other genera produce toxins that, in turn, accumulate in plants and lead to many problems, starting with the germination inhibition and disturbances in physiological processes [1,2,3,4]. Mycotoxins are toxic secondary metabolites produced by pathogenic fungi (not mushrooms) that accumulate in plant crops, which are toxic for animals and humans [5,6,7]. Some mycotoxins could induce liver cancer, and cancer of the nervous system [8]. Among toxic mycotoxins, *Aflatoxins* are the most common mycotoxin in poultry feed; they are quite stable and resistant to degradation. Aflatoxins are poisonous, carcinogenic, mutagenic, immunosuppressive, and teratogenic secondary metabolites, formed by *Aspergillus flavus*, *A. parasiticus* [9], and *A. nomius* [10]. Common groups of aflatoxins are: aflatoxin B_1_ (AFB_1_), aflatoxin B_2_ (AFB_2_), aflatoxin G_1_ (AFG_1_) and aflatoxin G_2_ (AFG_2_) [11,12]. Furthermore, the most toxic of these aflatoxins is AFB_1_ [13,14]. AFB_1_ may be converted into its hydroxylated form, called aflatoxin M_1_ (AFM_1_), which is excreted in the breast milk of humans and animals, following the ingestion of AFB_1_-contaminated food or feed [15]. In developing countries, about 4.5 billion people are chronically exposed to uncontrolled amounts of aflatoxins [16]. Consumption of contaminated products causes aflatoxicosis in humans and animals. Aflatoxicosis may be acute and chronic. The acute condition causes death, while the chronic condition results in immune suppression and cancer. In humans, it is characterized by vomiting, abdominal pain, pulmonary edema, convulsions, coma, and death, with cerebral edema and fatty involvement of the liver, kidneys, and heart [17]. In plants, the action of mycotoxins is represented in affecting the permeability of the cell membrane or by inhibiting the enzymatic activity in the plant and, thus, disturbances occur in the enzymatic reactions [18]. The production of mycotoxins also alters the plant growth, yield and their primary and secondary metabolism [19]. The accumulation of aflatoxins in maize is a destructive agricultural problem for human and animal health, besides the loss of yield [20]. For instance, a clear decline in plant physiological activity and chlorophyll content were reported in plants in response to *A. flavus* infection [21]. Further, many fungal pathogens produce toxins that inhibit photosynthesis by decreasing chlorophyll biosynthesis and inhibiting photosynthetic key enzymes [22]. The fungal infection leads to an imbalance in the transport of nutrients and water within the plant organs. Consequently this disturbs plant functions, causing disease in the entire plant [23]. Furthermore, infection with *A. flavus* alters the internal structures of the affected tissues, leading to an abnormal increase or decrease in organelles size [24]. For instance, infection with *A. flavus* directly affects the protoplast of the host cells that destroy or kill the cells [25].

Although the use of chemical pesticides in combating plant infection with pathogenic fungi that causes plant diseases has generally been proven to have tangible efficiency, it has negative effects on soil, plants and microorganisms [26]. The accumulation of chemical pesticides in plants can indirectly affect human and animal health [27]. Moreover, they lead to an increase in production costs, while not ensuring the effectiveness of their impact [28]. All of these reasons led to the transformation of the concept of agricultural sustainability with the need to improve the product quality and freedom from pesticide residues and toxicity [29]. In this context, the attention of scientists has turned to the use of derived biocides, such as endophytic fungi from microorganisms or natural extracts against plant pathogens [30]. This increases plant resistance by preventing or limiting the progression of damage under biotic or abiotic stress [31]. Endophytic fungi are fungi that can grow in healthy plant tissues without any harmful effects on their host plants [32,33]. In general, leaves are the main source of the endophytic fungal community, promoting the effect on plant health [34]. The degree of microbial diversity of living host plants differs according to the plant species that have been described as protecting agents against biotic attacks [35]. In this regard, many studies reported that endophytic fungi are rich in secondary metabolites, with a broad range of biological activities, such as antifungal activity [32,33]. The aim of this study was to mitigate the effect of *A. flavus* infection and its mycotoxins on maize growth, anatomy, and the redox status of maize seedlings, using the endophytic *A. fumigatus*. This will clear the way for alternative and eco-friendly methods for managing fungal pathogens.

## 2. Materials and Methods

### 2.1. Chemical Reagents

Sodium hypochlorite NaOCl (Sigma-Aldrich, Germany), potato dextrose agar (PDA) medium (Oxoid, Basingstoke, UK) (Sigma-Aldrich), PD broth medium (Sigma-Aldrich), chloramphenicol (Sigma-Aldrich), ethanol (Sigma-Aldrich), tween 80 (Sigma-Aldrich), acetone, 5% trichloroacetic acid, thiobarbituric acid (TBA), titanium dioxide, sulfuric acid, folin reagent, sodium carbonate Na_2_CO_3_, sulfosalicylic acid, ninhydrin acid, phosphoric acid, glacial acetic acid, phosphate buffer pH 6.8, phosphate buffer (pH 7.8), pyrogallol, hydrochloric acid HCl, phosphate buffer pH 7, and hydrogen peroxide H_2_O_2_ were used in this study.

### 2.2. Isolation of FUNGAL Pathogen

Isolation of the fungal pathogen *A. flavus* was carried out by minor modification of the method from [36]; therefore, 100 infected corn seeds (Giza-Balady) were surface sterilized in NaOCl (2.5% for 60 s) followed by washing with sterile distilled water. The sterilized seeds were cultured on potato dextrose agar (PDA) medium (Oxoid) supplemented with 2% chloramphenicol (Sigma-Aldrich). Cultivated plates were incubated for 4 days at 27 °C and examined daily. Growing mycelium was sub-cultured at 27 °C for 5 days on PDA medium [37,38].

### 2.3. Isolation of Endophytic Fungi

Isolation of endophytic fungi was achieved using the method of Aldinary et al. [35]. Apparently healthy *Moringa oleifera* leaves were obtained from the National Research Center, Dokki, Egypt. These leaves were washed with tap water then sterilized with 70% ethanol for 1 min, then with 4% NaOCl for 1 min. The epiphytic sterilized leaves were cultivated on PDA supplemented with chloramphenicol (0.2 g/L). The cultivated plates were incubated at 27 °C ± 2 for 3 weeks through daily examination, then sub-cultured into a sterilized PDA medium.

### 2.4. Morphological and Molecular Identification of Fungal Pathogen and Endophytes

Morphological identification of the target fungus was carried out by observing the morphological characteristics (color, texture, and appearance) and microscopic characteristics using light and scanning electron microscope (SEM) [5,39,40,41]. Molecular identification of fungal pathogen and fungal endophyte was carried out using internal transcribed spacer (ITS) genes according to Khalil et al. [32]. DNA was extracted from agar cultures using Quick-DNA Fungal/Bacterial Microprep Kit (Zymo research; D6007, Irvine, CA, USA) following the manufacturer’s protocol and supported by Sigma Scientific Services Company (Giza, Egypt). PCR was performed using Maxima Hot Start PCR Master Mix (Thermo, Waltham, MA, USA; K1051). The primers used were Forward ITS1-F (50-TCCGTAGGTGAACCTGCGG-30) and Reverse ITS4-R (50-TCCTCCGCTTATTGATATGC-30). The reaction conditions were: initial denaturation at 95 °C for 10 min, followed by 35 cycles of denaturation at 95 °C for 30 s, annealing at 57 °C for 30 s and extension at 72 °C for 1.5 min; a final extension phase was performed at 72 °C for 10 min. At first, the following components were added for each 50 μL total reaction volume at room temperature: Maxima Hot Start PCR Master Mix (2×) 25 μL, ITS1 Forward primer 1 μL (20 μM), ITS4 Reverse primer 1 μL (20 μM), Template DNA 5 μL, water, and nuclease-free 18 μL. The obtained PCR product was purified using GeneJET PCR Purification Kit (Thermo K0701) following the manufacturer’s protocol. Finally, sequencing to the PCR product in GATC Company (Konstanz, Germany) by use of an ABI 3730xl DNA-sequencer was performed using the same forward and reverse primers mentioned above and by combining Sanger and 454 technologies for DNA sequencing according to manufacturer’s instructions. The obtained ITS sequences were aligned by Clustal W (codons) with the required minor manual adjustments. The final sequence was compared with similar sequences retrieved from DNA databases by using the NCBI n-BLAST search program in the National Center for Biotechnology Information (NCBI). Evolutionary analyses were conducted in Molecular Evolutionary Genetics Analysis MEGA-X.

### 2.5. In-Vitro Antifungal Activity

Two isolated fungal endophytes, i.e., *A. fumigatus* and *A. terreus,* were evaluated as antifungal against toxigenic *A. flavus* using dual culture technique according to Singh and Sati [42]. The mycelial growth inhibition percentage was calculated according to the following formula:

I (%) = (Dc − Dt/Dc) × 100

I (%): inhibition percentage; Dc: average diameter of the control colonies; Dt: average diameter of the treated colonies.

To inhibit aflatoxins production, PD broth medium was inoculated with fungal endophyte (*A. fumigatus*) and fungal pathogen (*A. flavus*), while control included the *A. flavus* only without treatment at the same conditions.

### 2.6. Extraction and Detection of Aflatoxins by High-Performance Liquid Chromatography (HPLC)

From fungal culture: AFs were extracted using liquid–liquid extraction method. Each sample was adjusted to pH 2 with HCl and an aliquot (5 mL) was transferred in a separating funnel. Next, 10 mL of dichloromethane was added three times and the mixture was shaken for 1 min, then the dichloromethane extracts were collected in a flask. The final extract was evaporated to dryness in a rotary evaporator at 35 °C. The residue was dissolved in 1 mL of H_2_O:CH_3_OH 1:1 for the HPLC.

From plant material: AFs were extracted using liquid–liquid extraction method. All samples were homogenized and stored in a refrigerator at −20 °C until extraction. Then, 1 g of each homogenized sample was placed in a centrifuge tube with 0.5 g of NaCl and 10 mL of extraction solution (CH_3_OH:CH_3_CN:H_2_O 10:45:45 *v*/*v*/*v* adjusted to pH 3 with o-phosphoric acid) was added. The mixture was shaken for 30 min in an ultrasonic bath and then centrifuged at 5000 rpm for 5 min. Solid phase extraction was performed using hydrophilic lipophilic balanced (HLB) copolymer cartridges (Polyntell AttractSPETM W/O 3 mL, 60 mg). First, 2 mL of clarified extract was diluted with 18 mL of HCl 0.01 N then passed through a conditioned cartridge (conditioning was made first with 3 mL of CH_3_OH followed by 3 mL of H_2_O). After a washing step with 3 mL of water, toxins were eluted with CH_3_OH:CH_3_CN 70:30 acidified with 0.1% of CH_3_COOH. The eluate was dried in vacuum concentrator (Eppendorf) at 30 °C, and the extract was then dissolved in 1 mL of H_2_O:CH_3_OH 1:1 for the HPLC [43,44]. The HPLC analysis was applied for quantification of all aflatoxins, which included AFG_1_, AFG_2_, AFB_1_ and AFB_2_. Concentration of AFs standard AFG_1_, AFG_2_, AFB_1_ and AFB_2_ were 50, 15, 50 and 15 ng/mL. The column used was Agilent C18 (4.6 mm × 250 mm i.d., 3.5 μm). The mobile phase was water:methanol:acetonitrile 60:30:10 and the flow rate was 1 mL/min. The injection volume was 20 μL for each of the sample solutions. The fluorescence detector was adjusted to 360/450 nm (Excitation/Emission). The column temperature was maintained at 40 °C. The limit of quantification (LOQ) for AFs is 0.01 ng/mL.

### 2.7. In-Vivo Pot Experiments

Pot experiment was conducted at the research garden of the Botany and Microbiology Department, Faculty of Science, Al-Azhar University, Egypt. Apparently healthy corn seeds (single hybrid Giza-162) were obtained from Agricultural Research Center (ARC), Giza, Egypt, and seeds were surface sterilized by immersing in 0.01% sodium hypochlorite for 60 s followed by 70% ethanol for 60 s, then washed with distilled water [45]. The method of Tédihou et al. [46] was used for inoculation of *A. flavus* culture which was originally isolated from infected maize seeds. After purification of *A*. *flavus* by single-spore isolation, the fungus was grown in plates containing PDA medium, then the plates were kept in an incubator at 28 °C in the dark for 7 days. The conidia were collected and suspended in sterile distilled water. Approximately 0.1 mL of tween 80 was added per liter of water and the concentration of the suspension was determined using a hemocytometer to 2.7 × 10^7^ conidia per liter. The inoculum was 20 mL per pot. The seeds were sown in plastic pots with a diameter of 15 cm containing 3 kg of clay soil. The sterilized seeds were divided into three groups: (1) Control group, in which the healthy seeds were soaked in distilled water for 2 h then sown in non-infected soil, (2) Control infected group, in which the healthy seeds were soaked in distilled water for 2 h then sown in infected soil with *A. flavus*, and (3) Infected group, where the seeds were soaked in *A. fumigatus* filtrate for 2 h then sown in infected soil. The replicates were 5 for each treatment. The three treatments for each pot meant that 8 seeds were planted 3 cm deep in each pot. Potted samples were harvested 14 days after seed germination. The inoculums (*A. flavus* and *A. fumigatus*) were prepared according to Attia et al. [47].

### 2.8. Photosynthetic Pigments

Photosynthetic pigments were determined according to Lichtenthaler and Buschmann [48]. A previously mentioned technique was used to measure the presence of chlorophyll a, chlorophyll b and carotenoids in fresh *Zea mays* leaves (three replicates for each treatment). Throughout this procedure, 100 mL of acetone (80%) was used for photosynthetic pigments extraction from fresh maize leaves (1.0 g) and the extract was filtered and the established green color was spectrophotometrically calculated at 665, 649 and 470 nm. Photosynthetic pigments were determined by the equations, chlorophyll (a) mg/g tissue = 11.63 (A665) − 2.39 (A649), chlorophyll (b) mg/g tissue = 20.11 (A649) − 5.18 (A665), chlorophyll (a + b) mg/g tissue = 6.45 (A665) + 17.72 (A649) and Carotenoids = 1000 × O.D_470_- 1.82C_a_ − 85.02C_b_/198 = mg/g fresh weight. “A” denotes the reading of optical density.

### 2.9. Lipid Peroxidation (MDA) and Hydrogen Peroxide (H_2_O_2_) Contents

The content of Malondialdehyde (MDA) in fresh *Zea mays* leaves (three replicates for each treatment) was assessed according to Hu et al. [49]. Fresh leaf samples (0.5 g) were extracted with 5% trichloroacetic acid and centrifuged at 4000× *g* for 10 min. Two milliliters of the extract was mixed with 2 mL of 0.6% thiobarbituric acid (TBA) solution then the reaction mixture was incubated in a water bath for 10 min. After cooling, the absorbance of the developed color was measured at 532, 600 and 450 nm. MDA content was determined using the following equation: 6.45 × (A532 − A600) − 0.56 × A450. The H_2_O_2_ content of fresh *Zea mays* leaf (three replicates for each treatment) was measured as stated by Mukherjee and Choudhuri [50]. In this method fresh leaves (0.5 g) were extracted in 4 mL of cold acetone then 3 mL of the extract was mixed with 1 mL of 0.1% titanium dioxide in 20% (*v*/*v*) sulfuric acid and the mixture was then centrifuged at 6000× *g* rpm for 15 min. The formed yellow color was measured at 415 nm.

### 2.10. Assessment of Proline Level and Phenolics

Total phenols content was estimated according to the method described by Dai et al. [51]. In this method, 1 g of dried shoots was extracted in 5–10 mL of 80% ethanol for at least 24 h. After centrifugation, the residue was re-extracted 3 times with 5–10 mL of 80% ethanol. Then, the clarified supernatants of the two extracts were filled to 50 mL with 80% ethanol. Following this, 0.5 mL of the extract was mixed well with 0.5 mL of folin’s reagent then shook for 3 min. One milliliter of saturated Na_2_CO_3_ solution and three milliliters of distilled water were well mixed with the extract. After 1 h, the blue color was measured at 725 nm. The method of Bates et al. [52] was used for estimation of proline. In this procedure, the dried shoots (0.5 g) were digested in 10 mL (3%) of sulfosalicylic acid. As such, 2 mL of filtrate reacted with 2 mL of ninhydrin acid (1.25 g ninhydrin in 30 mL of glacial acetic acid and 20 mL of 6 M phosphoric acid) and 2 mL of glacial acetic acid in a boiling water bath for 1 h, then the reaction was stopped by placing the reaction mixture in an ice bath. We added 4 mL of toluene to the mixture, then read the absorbance at 520 nm. Three replicates for each treatment in biochemical indicators were carried out. Proline was determined according to standard curve and the following equation:

mg/g proline = ((X) ppm × mL extract)/(2X sample dry weight × 100)

### 2.11. Assessment of Antioxidant Enzymes

The activity of antioxidant enzymes, i.e., antioxidant enzymes peroxidase (POD), polyphenol oxidase (PPO), superoxide dismutase (SOD) and catalase (CAT)) in fresh shoots were determined according to Bergmeyer [53], Dai et al. [51], Kong et al. [54] and Chen et al. [55]. Superoxide dismutase (SOD) were peroxidase (POD), catalase and polyphenol oxidase (PPO) enzymes were estimated in plant materials. The enzyme extract was obtained as the follows: 2 g of the terminal buds in addition to the first and second young leaves 14 days after seed germination were homogenized with 10 mL of phosphate buffer pH 6.8, then centrifuged at 2 °C for 20 min at 20,000 rpm, after which the clear supernatant was taken and enzyme activity was determined. The activities were determined by equation (A × T v × 60 min)/(t × v × F. Wt.), where A is the absorbance of the sample after incubation minus the absorbance at zero time, T v is the total volume of filtrate, t is the time (minutes) of incubation with substrate and v is the total volume of filtrate taken for incubation and F. Wt. is the fresh weight used.

#### 2.11.1. Superoxide Dismutase (SOD) Activities

The solution (10 mL) consisted of 3.6 mL of distilled water, 0.1 mL of enzyme, 5.5 mL of 50 mM phosphate buffer (pH 7.8) and 0.8 mL of 3 mM pyrogallol (dissolved in 10 mM HCl), used for determination of SOD. The rate of pyrogallol reduction was measured at 325 nm with UV spectrophotometer (Jenway).

#### 2.11.2. Peroxidase (POD) Activities

The solution containing 5.8 mL of 50 mM phosphate buffer pH 7, 0.2 mL of the enzyme extract and 2 mL of 20 mM H_2_O_2_ after addition of 2 mL of 20 mM pyrogallol was used for determination of POD. The rate of increase in absorbance as pyrogallol was determined spectrophotometrically by UV spectrophotometer (Jenway) within 60 s at 470 nm and 25 °C.

#### 2.11.3. Polyphenol Oxidase (PPO) Activities

The 125 µmol solution of phosphate buffer (pH 6.8), 100 µmol pyrogallol, and 2 mL of enzyme extract were used for determination of PPO. After the incubation period of 5 min at 25 °C, the reaction was stopped by adding 1 mL 5% H_2_SO_4_. The blank sample was made by using very-well-boiled enzyme extract and the developed color was measured at 430 nm.

### 2.12. Proportion of Mycotoxins

Three biological replicates of the three treatments were dried to determine the proportion of mycotoxins in grains according to F Abdallah et al. [56].

### 2.13. Microscopic Examination

Ultra-structural variations generated in leaves and roots were examined (three replicates for each treatment) with a JOEL JM 100-C Transmission Electron Microscope (Electron Microscope Unit, Regional Center of Mycology and Biotechnology, Al-Azhar University, Cairo, Egypt). The chosen samples were fixed and handled for electron microscopy examination [57]. Small sections of leaf and root specimens were individually cut and fixed in Universal E.m. fixative and kept at 4 °C till processing. Samples were rinsed twice in 0.1 M phosphate buffer for 10 min. Post fixation in 1% of 0.2 M phosphate-buffered osmium tetra-oxide for 1 h at 4 °C. Then samples were re-rinsed with phosphate buffer for 10 min, after which dehydration started. As such, 50, 70, and 95% ethyl alcohol was added twice for 15 min with continuous shaking. Propylene oxide was added twice for 8 min, and then mixed with epon 1:1 mixture for 30 min, then to 1:3 ratio mixture for 30 min each time with continuous shaking. Vials were opened and placed in individual beakers and left overnight to allow for the evaporation of propylene oxide. The next day, each piece of tissue was embedded in individually labeled beam capsules. Blocks were trimmed to trapezoid shape. Thin sections were made using an LKB ultra-microtome and 3–4 grids were loaded for each specimen and kept in a Petri dish with proper identification. The ultrathin sections were stained for 10 min in a mixture of saturated solution of uranyl acetate and acetone (in equal volume), then with Reynold’s lead citrate. Two grids were loaded for each specimen and kept in a Petri dish with proper identification.

### 2.14. Statistical Analysis

One-way analysis of variance (ANOVA) was applied to the resulted data. Duncan’s multiple range test using Costate (Cohort’s software, Monterey, CA, USA) was applied to show statistically significant differences among the treatments at *p* < 0.05. Obtained data were shown as means ± standard errors (*n* = 3).

## 3. Results and Discussion

### 3.1. Isolation and Identification of Fungal Pathogens

**Figure 1:**
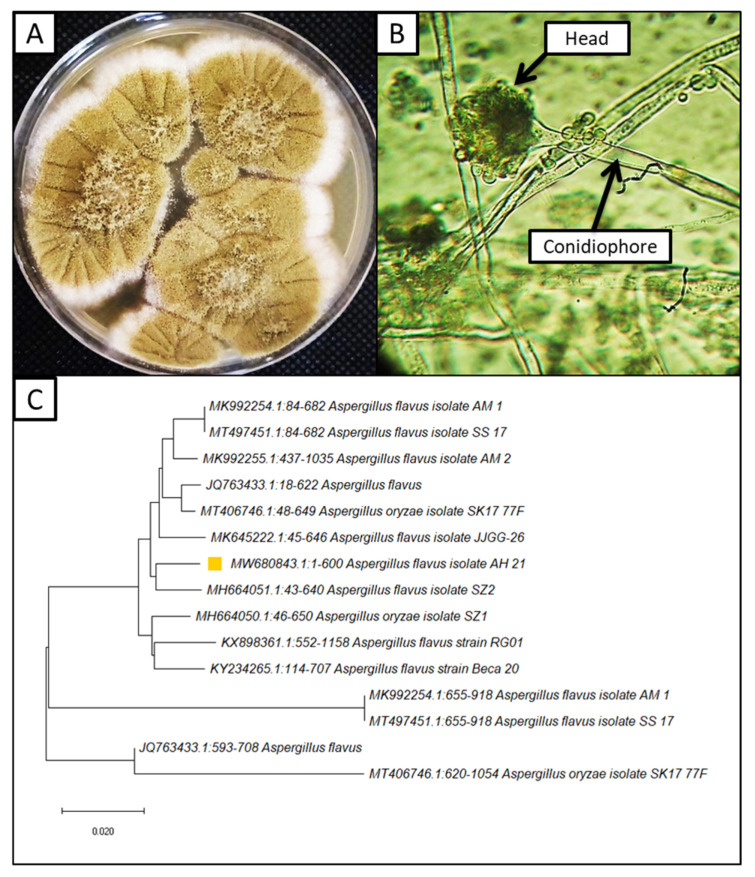
Morphological and molecular identification of *A. flavus* (**A**–**C**); (**A**) surface of culture on PDA media; (**B**) conidiophores and head under the light microscope (400×); (**C**) phylogenetic tree for *A. flavus* (orange square).

The fungal pathogen was isolated from infected corn seeds on a PDA medium morphologically, and fungal isolates were identified as *A. flavus.* Observed colonies on the PDA were 40 mm in diameter at 27 °C after 4 days of growing (Figure 1A). They often display central floccose, white, conidial heads, usually borne uniformly over the whole colony. Characteristically, the colony color is greyish green, yellow green, then becoming greenish in old age. Vesicles globose to sub-globose shape, metula and phialides long covered 75.00% of the head; conidia were spherical to sub spheroidal, with relatively thin walls (Figure 1B). To validate the morphological identification, molecular identification was performed. It confirmed the morphological identification, where the isolated strain is similar to *A. flavus,* with an identity of 97.50% (Figure 1C). Furthermore, this strain was deposited in GenBank with the accession number MW680843. The isolation of *A. flavus* from infected corn plants was in line with previous studies [58,59].

### 3.2. Isolation and Identification of Endophytic Fungi

**Figure 2:**
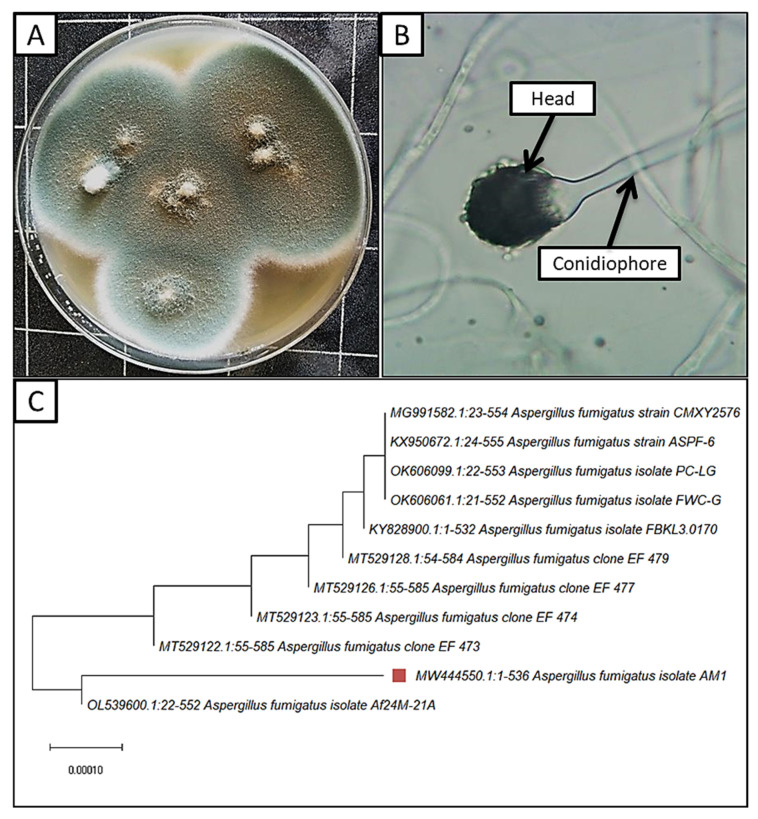
Morphological and molecular identification of *A. fumigatus* (**A**–**C**); (**A**) surface of culture on PDA; (**B**) conidiophores and head under the light microscope (400×); (**C**) Phylogenetic tree for *A. fumigatus* (brown square).

Two fungal isolates were isolated from *Moringa oleifera* leaves, which were morphologically identified as *A. fumigatus* and *A. terreus*. According to [33,60] the morphological identification, colonies grow rapidly on a PDA medium, reaching 60 mm in diameter at 27 °C after 4 days (Figure 2A). The rate of growth is rapid, with a smoky-grayish-green color and pale-yellow reverse color. Conidiophores ending with oval vesicle bearing a single series of sterigmata covered almost half of the vesicle. The sterigmata bore a series of sub-spherical or oval, rough-walled conidia, and the conidial head was a columnar shape (Figure 2B). Molecular identification also confirmed the morphological identification as *A. fumigatus* AM1, with a similarity percentage of 98.50%. *A. fumigatus* AM1 was recorded in GenBank with the accession number MW444550. Previous studies revealed that *A. fumigatus* can be isolated from healthy *Moringa oleifera* leaves [61,62]. *A. fumigatus* was selected for further study, according to the noticeable antagonistic effect against *A. flavus*.

### 3.3. Antagonistic Effect of Endophytic A. fumigatus and A. terreus against A. flavus

**Figure 3:**
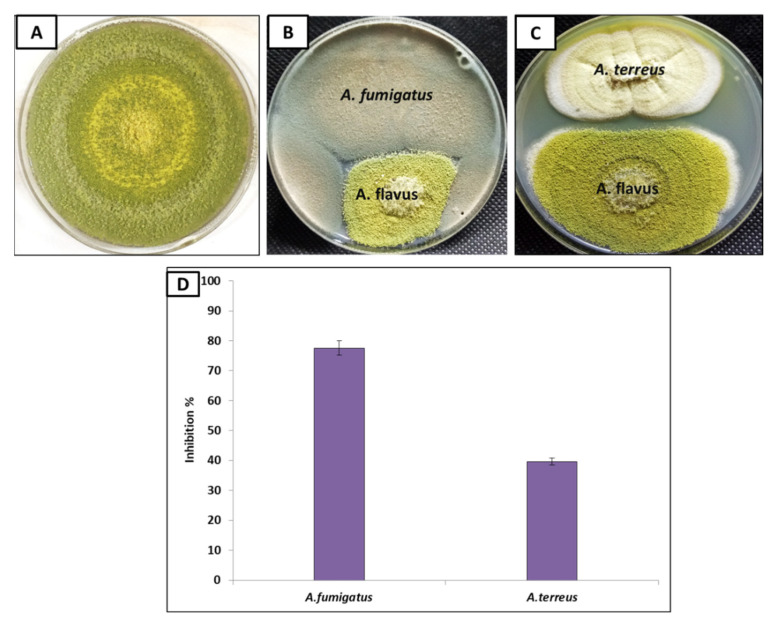
Dual culture of endophytic *A*. *fumigatus* (**B**) and *A. terreus* (**C**) against *A. flavus*, compared to *A. flavus* only; control (**A**); the inhibition percentage of growth for each endophyte was shown in (**D**).

Fungi have the ability to perform mycoparasite interaction with other fungi, where they can produce secondary metabolites that could control the growth of other pathogenic fungi [63,64]. Consequentially, fungal endophytes *A. fumigatus* and *A. terreus* were used to inhibit the growth of aflatoxin, producing *A. flavus* (Figure 3B,C). The results revealed that both *A*. *fumigatus* and *A. terreus* reduced *A. flavus* growth, where *A. fumigatus* was more efficient than *A. terreus*. Moreover, the inhibition percentage of *A. fumigatus* against *A. flavus* was 77.60%, while in the case of *A. terreus,* it was 39.70% (Figure 3D). Therefore, fungal endophyte *A. fumigatus* was selected for further experiments.

### 3.4. In-Vitro Inhibition of Aflatoxins Production of A. flavus Using A. fumigatus in Liquid Medium

**Figure 4:**
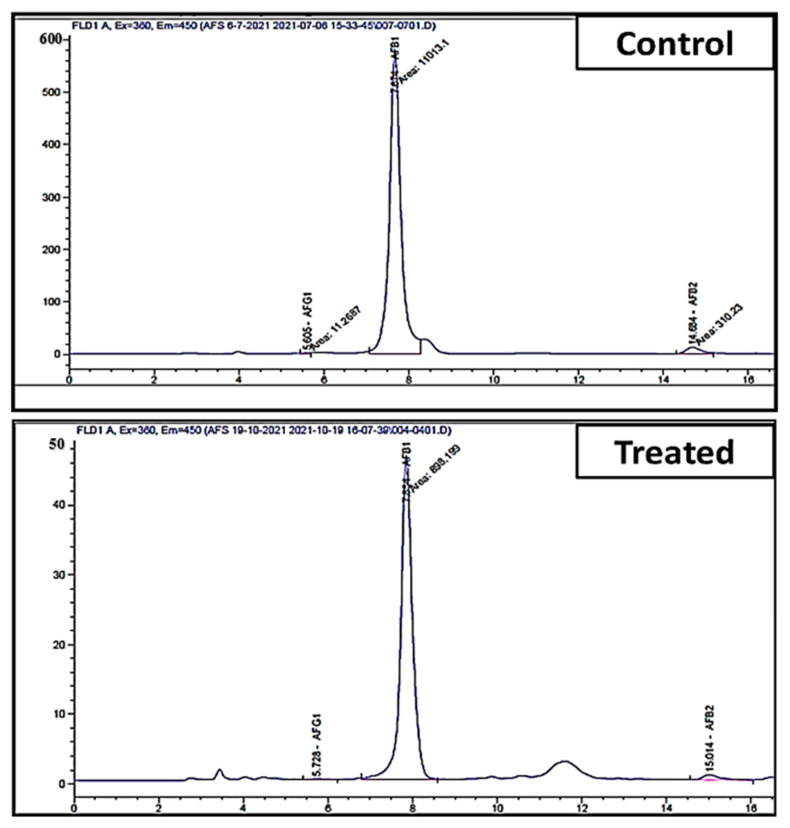
HPLC chromatogram of aflatoxin level in control (infected maize with *A. flavus*) and treated (infected maize treated with endophytic *A. fumigatus*).

In vitro, the effect of *A. fumigatus* on the production of aflatoxins by *A. flavus* in a liquid medium was measured using HPLC methods (Figure 4). The results revealed that *A. fumigatus* reduced all aflatoxin types in the medium, reducing AFG_1_ from 6.88 to 1.40 ng/mL with 79.60% inhibition, and also reduced AFB_1_ from 3647.1 to 332.5 ng/mL with 90.90% inhibition. The fungal endophyte *A. fumigatus* has great efficiency in decreasing the most toxic and commonly occurring toxin (AFB_1_), which has been classified as a group Ι human carcinogen by the International Agency for Research on Cancer [65]. Furthermore, *A. fumigatus* reduced the production of AFB_2_ from 99.39 to 6.80 ng/mL, with a high inhibition value of 93.10% (Table 1). On the other hand, AFG_2_ was not detected, neither in the control nor treated samples.

### 3.5. In-Vivo Inhibition of Aflatoxins Production in Infected Corn Plants

**Figure 5:**
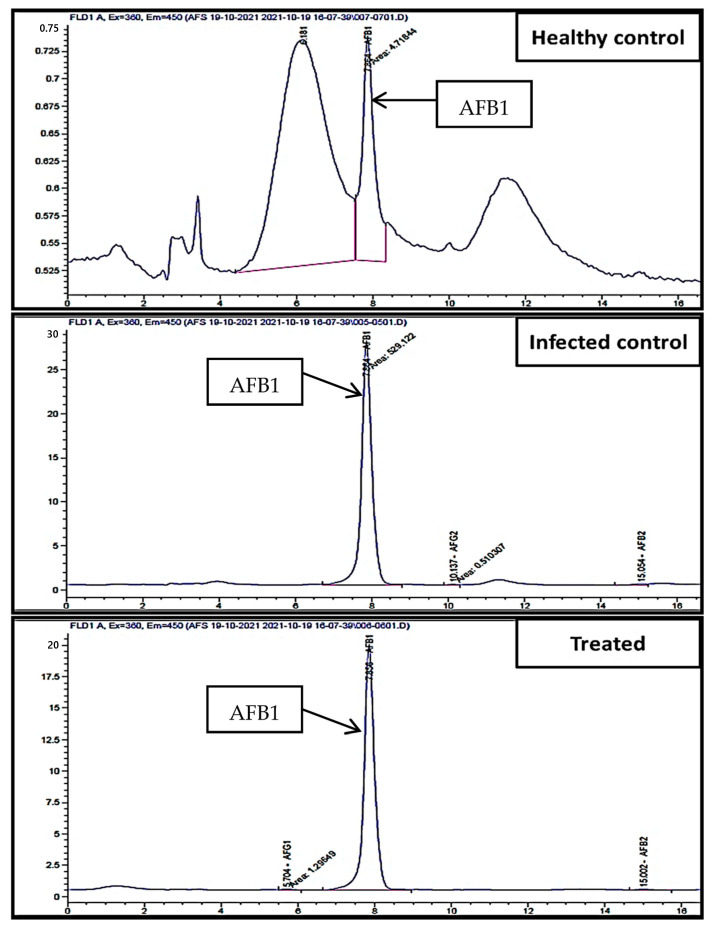
HPLC chromatogram of healthy and infected control and treated samples in in-vivo maize seedlings.

*A. flavus* induced mycotoxins in plant grains, making them unsuitable for human and animal consumption [64]. Therefore, the prevention of pre and postharvest mycotoxins, particularly in foods, is urgently required. The results in Table 2 and Figure 5 showed that the *A. flavus* pathogen is considered to be a high aflatoxins producer, where the most produced aflatoxins were AFB_1_, then AFB_2_ and AFG_2_ type (497.09, 4.37 and 1.10 ng/g, respectively); however, AFG_1_ was not detected. Similarly, Savić et al. [66] reported that *A. flavus* was the main source of aflatoxins B in maize plants. These findings were also confirmed by other studies [67,68]. Interstingly, *A. fumigatus* reduced all aflatoxins types in the corn plant, while it completely prevented AFG_2_ production but reduced AFB_1_ from 794.09 to 462.57 ng/mL, with an inhibition percentage of 41.74%. It also reduced aflatoxin AFB_2_ from 4.37 to 1.63 ng/mL, with an inhibition percentage of 62.70%. In line with our findings, Abbas et al. [69] reported that non-mycotoxigenic fungi can control the aflatoxins produced by toxogenic *A. flavus* in corn plants. These results are explained by the fact that *A. fumigatus* can degrade aflatoxin groups and metabolize to less toxic, or nontoxic, components [70] or by increased nutrient competition [71]. This can also be explained by the ability of endophytic *A. fumigatus* to induce high activities of POD, PPO, SOD and CAT enzymes in treated maize plants that support the plant to mitigate aflatoxins-induced oxidative stress [72].

### 3.6. A. fumigatus Attenuates Inhibitions of Maize Seedling Germination and Growth Caused by A. flavus

**Figure 6:**
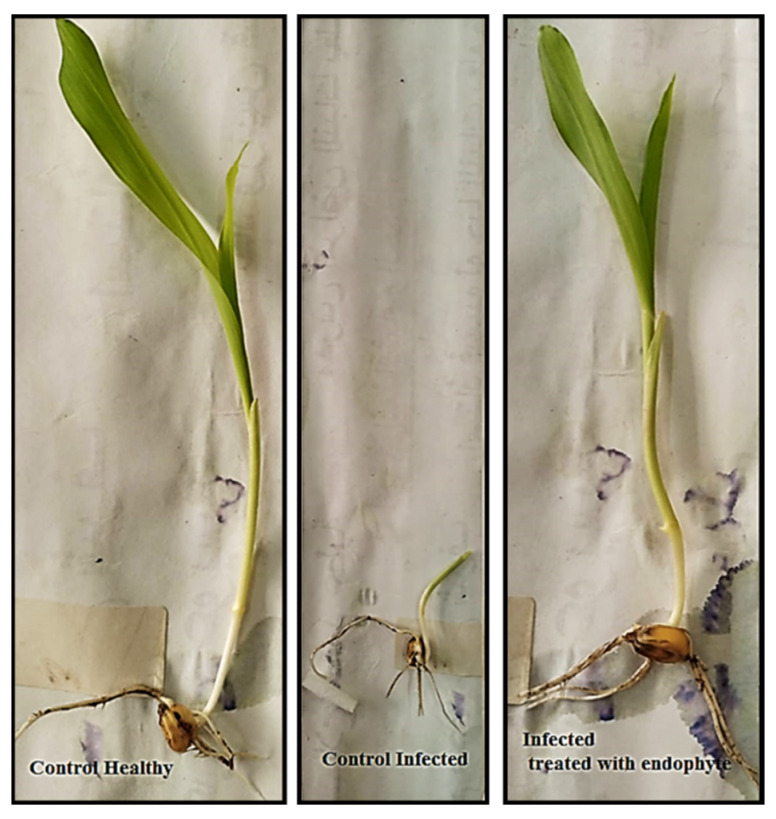
Growth characteristics of corn seedlings in different treatments: control healthy, control infected and infected treated with endophytic fungus *A. fumigatus.*

A noticeable decrease in the percentage of maize seeds germination, as well as a decline in the shoot and root lengths of maize seedlings, were recorded for *A. flavus*-treated maize plants (Table 3 and Figure 6). In this regard, the germination % was decreased by 53.2%, and both stem and root lengths were decreased by 56.00% and 67.60%, respectively, compared to healthy seedlings. Similarly, the infection of corn seeds with the toxic fungus *A. flavus* leads to a significant inhibition in the germination process [73]. In this context, the infection of seeds with pathogenic fungi leads to seed abort and necrosis, reduction in seed germination, seedling damage and plant disease induction [73]. This decrease was likely due to the secretion of mycotoxins, which impede the germination process, possibly by affecting seed respiration [74]. In line with our results, aflatoxins produced by pathogenic fungi reduced the seed germination and seedling growth of corn and bean plants [75]. Moreover, according to Bhat, Fazal [76], and El-Naghy et al. [75], *A. flavus* reduced corn seed germination, metabolism, and seedling growth.

On the other hand, our results showed an increase in the seeds germination rate after endophytic *A. fumigatus* treatment, reaching 76.66%, with a recovery rate of 88.53%. *A. fumigatus* showed anti-fungal effects and inhibitory effects on mycotoxins production [77]. It is also considered as one of the growth-stimulating organisms that have the ability to produce natural hormones and vitamins that stimulate seed germination and plant growth [78,79]. It also stimulated phenols production and antioxidant enzymes activity, as well as the synthesis of antimicrobial phytoalexins [80,81].

### 3.7. Effect of A. fumigatus on Photosynthetic Pigments of Zea maize Seedlings

**Figure 7:**
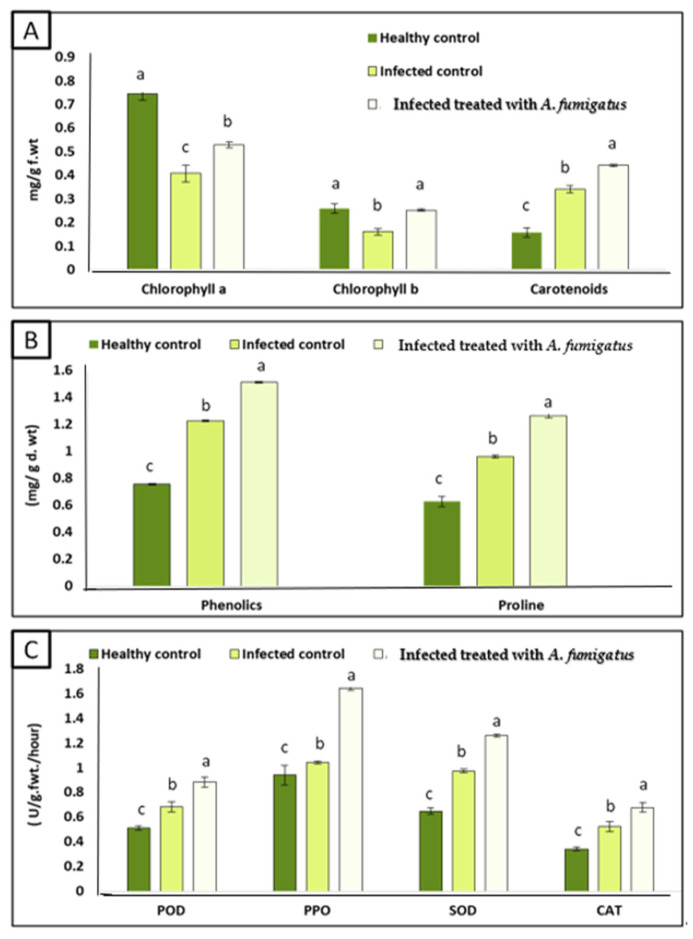
Effect of *A. fumigatus* on (**A**) photosynthetic pigments (chlorophyll a, chlorophyll b and carotenoids) (mg/g fresh weight), (**B**) phenolics, proline (mg/g dry weight) and antioxidant enzymes (**C**) activity (POD, PPO, SOD, and CAT) (unit/g fresh weight/hour). Data are expressed as means ± standard deviations of triplicate assays. The different alphabetic superscripts in each item are significantly different (*p* < 0.05).

The infection with *A. flavus* led to a noticeable decrease in the level of chlorophyll a and b, where the decrease in chlorophyll a and b contents reached up to 45.90% and 37.90%, respectively (Figure 7A). On the other hand, carotene pigments were increased in infected maize seedlings by 53.80%. These reductions can be attributed to the disruption of the photosynthesis process by pathogenic *A. flavus* infection, as it induced a disturbance in chloroplast structure and function [82]. The production of toxins can suppress the photosynthetic machinery and activity [83]. Possibly, *A. flavus* acts as biotic stress and significantly decreases the chlorophyll biosynthesis [21]. Our results are in line with the findings of Georgieva [84], who reported that the content of chlorophyll pigments reduced due to fungal infection. Interestingly, the application of *A. fumigatus* to infected plants stimulated the photosynthesis process, as indicated by the significant improvement in chlorophyll a and b, and this is strong evidence of the plants recovering from infection. The improvement role of endophytic fungi may be recognized as the fact that they stimulate the biogenesis of phytohormones and chlorophyll enzymes under different stressful conditions [85].

### 3.8. Effect of A. fumigatus on Stress Biomarkers

**Figure 8:**
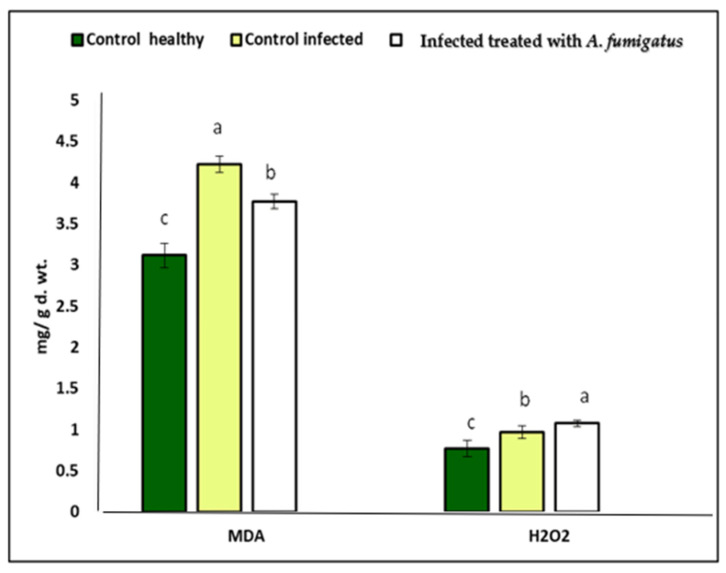
Effect of *A. flavus* and treatment with of *A. fumigatus* on (MDA) mg/g. dry weight and (H_2_O_2_) mg/g. dry weight of Maize seedlings. Data are expressed as means ± standard deviations of triplicate assays. The different alphabetic superscripts in the same column are significantly different (*p* < 0.05) means highly significant.

Oxidative stress caused by infection with *A. flavus* led to a serious disturbance in plant cells and raised the production of H_2_O_2_, as well lipid peroxidation (MDA), in the leaves of corn plants. Here, the infection with *A. flavus* led to increased oxidative stress markers, i.e., MDA and H_2_O_2_ (35.36 and 40.2%, respectively), compared to uninfected corn plants (Figure 8). On the other hand, the content of MDA and H_2_O_2_ declined in response to *A. fumigatus* treatment by 10.66% and 10.18%, respectively, compared to infected corn plants (Figure 8). In this regard, the application of *A. fumigatus* reduced oxidative damage by inducing antioxidant properties that scavenge ROS and prevent oxidative stress from affecting the cellular membranes [86,87].

### 3.9. Improvement in Redox Status by Treatment with A. fumigatus

To understand how plant-mitigated infection induced oxidative stress, antioxidant phenolics and proline were measured (Figure 7B). Maize seedlings grown in soil infected with *A. flavus* showed a significant increase in the content of phenols and proline (66.00%, 34.95%, and 180.00%). In agreement, Sultana et al. [21] found an increase in the total phenols and proline of rice plants infected with *A. flavus*. Mostly, the production of phenols increased in biotic stressed plant tissues [88]. Phenols play an important dual role in both repelling and attracting different organisms in the vicinity of plants [89]. The application of endophytic *A. fumigatus* enhanced proline and phenol contents in corn plants. These results are in harmony with [90,91]. Phenols act as protective agents by increasing plant immunity and inhibitors against fungal pathogens [92]. Both phenols and proline are involved in regulating and strengthening plant physiological immunity [93]. Proline is also increased by stress to maintain oxidative stress balance, plant cell wall stability, enzyme action, and capturing free radical ROS [94]. *A. fumigatus* application further increased the content of phenolics and proline by 23.30 and 29.97%, respectively. However, the content of proline was increased due to its role in osmoregulation and ROS scavenging [95]. Overall, the inoculation of maize plants with endophyte fungi is known to reduced oxidative stress by inducing the antioxidant metabolites and enzymes production [90].

At antioxidant enzymes activity level, our results, in Figure 7C, showed an increase in the activity of antioxidant enzymes (POD, PPO, SOD and CAT) in infected maize seedlings, where increases of 32.80%, 10.80%, 51.50%, and 53.8%, respectively, were observed. These results can be explained as a natural reaction to detoxify free radicals (ROS) [35]. These enzymes act as the initial steps in increasing plant resistance to various stresses, as well as the formation of phenolic compounds [96]. Antioxidant enzymes SOD, CAT, POD, and POO provide a large number of defensive enzymes associated with fungal infection [35]. POD, PPO, SOD, and CAT are defensive enzymes associated with mitigating biotic-stress-induced oxidative damage [97]. On the other hand, treatment with *A. fumigatus* showed a clear response by increasing the antioxidant enzymes of infected plants (POD, PPO, SOD and CAT), where the increases were 29.40%, 57.60%, 28.70%, and 30.10%, respectively. These increases in antioxidant enzymes by *A. fumigatus* are a way to protect cells from oxidative stress as a result of injury [35].

### 3.10. Ultra-Structural Study

**Figure 9:**
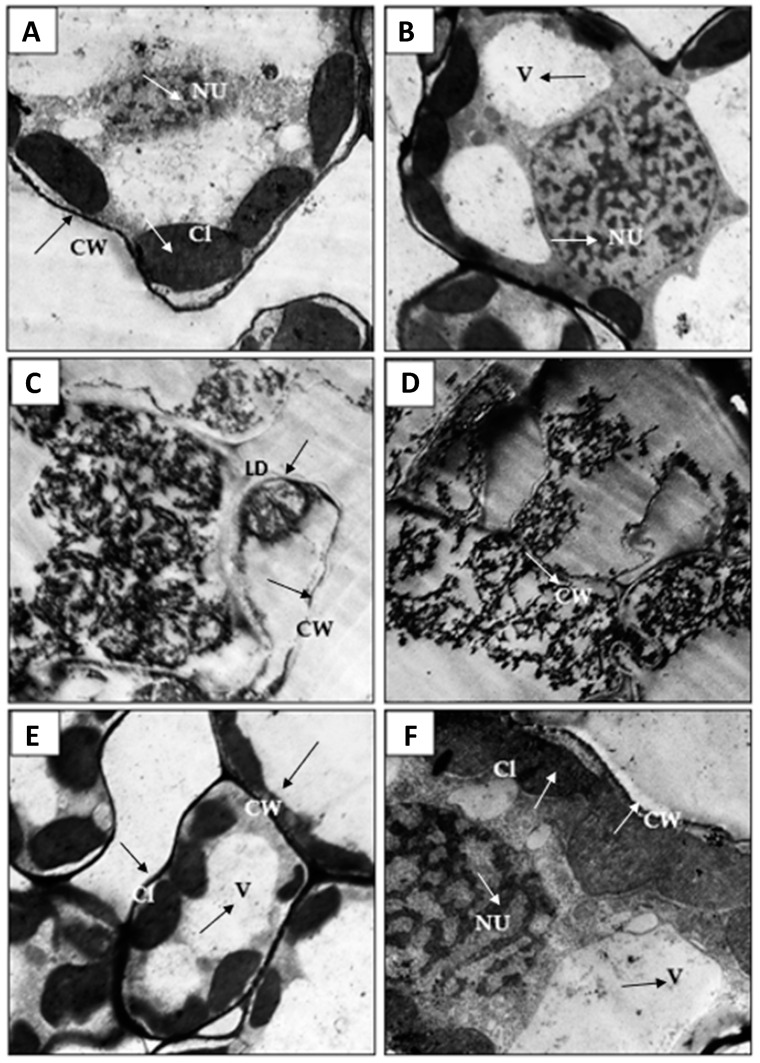
Ultra-micrograph sections of corn (*Zea mays* L.) showing leave cells, healthy (**A,B**), where thickened cell wall (CW) is clear compared with untreated, infected. There was a noticeable lack in the differentiation (LD) of the cells, cracking, and lysis in the cell wall and destruction in the nucleus (**C,D**); also, a presence of thickening in the cell wall (CW) was noticed in treated sample (**E**,**F**).

The ultra-structural features of corn leaves and roots of healthy and *A. flavus*-infected leaves were investigated using the ultra-thin section technique. The study aimed to detect the cytopathic effects of *A. flavus* on the cellular features of infected cells. By examination of ultrathin sections of healthy leaves, we found that the cells were of normal size, surrounded by a regular and thickened cell wall, and contained the nucleus in a normal shape (Figure 9A,B). Chloroplasts were located near the cell wall between the cytoplasm and tonoplast membrane and contained starch-encapsulated grains that contained grana grains. The mitochondria were arranged in regular rows and connected to each other by lamella granules.

On the other hand, infection induced severe disturbances, found in the internal structures after *A. flavus* infection (Figure 9C,D). There was a lack in the differentiation of the cells, cracking, and lysis in the cell wall, destruction in the nucleus, and semi-lysis of chloroplasts, and these notes are consistent with Ismaiel and Tharwat [98]. *A. flavus* caused soft rot diseases by forming toxins or by producing enzymes that break down plant cell walls [99]. Soft rot is described as the complete decomposition of plant tissues by the pathogen, where the plant tissue becomes soft and gelatinous in texture and accompanied by an unpleasant odor sometimes [100]. Interestingly, by examining the leaves of plants treated with *A. fumigatus*, a recovery was observed in the anatomical structures, where healthy medium-sized cells and chloroplasts containing starch granules coated with grana near the cell wall were observed. Further, the presence of thickening in the cell wall was found (Figure 9E,F).

**Figure 10:**
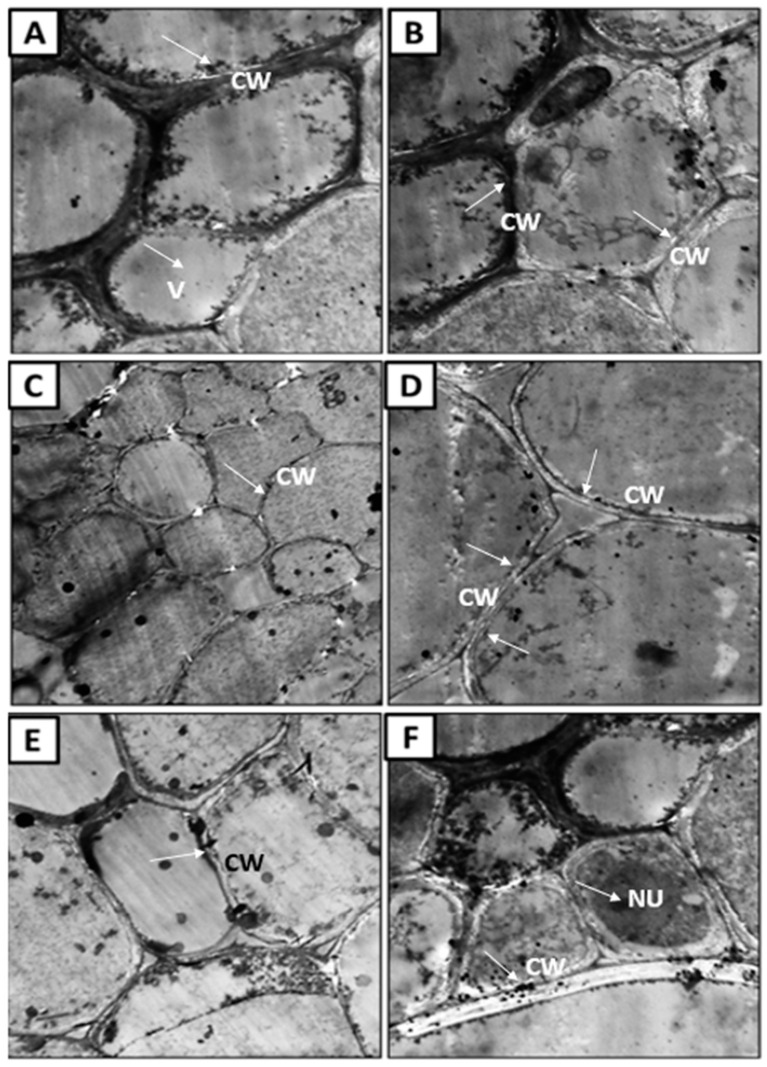
Ultra-micrograph section of corn (*Zea mays* L.) showing healthy root cells (**A,B**), where thickened cell wall (CW) is observed compared with untreated, infected sample. There was a noticeable lack in the differentiation (LD) of the cells, cracking, and lysis in the cell wall and destruction in the nucleus (**C,D**); also, a presence of thickening in the cell wall (CW) was noticed in treated sample (**E,F**).

At the root level, healthy plant roots showed a regular, cohesive, and thickened cell wall that contained cytoplasm inside the nucleus, mitochondria, and the rest of the cell’s organelles were not affected (Figure 10A,B). On the contrary, it was found that the cell wall of the infected plants was broken and irregular (Figure 10C,D), indicating the lack of coherence and harmony of cell organelles, the decomposition of the nucleus and the disappearance of the cytoplasm. These pathological observations agreed with [101,102], who found that cells of infected plants show elongated deformation and decomposition of the nucleus. Further, in the roots of plants treated with *A. fumigatus* (Figure 10E,F), many differences were observed for the infected plant, where a cohesive and thickened cell wall and the presence of its nucleus inside the cytoplasm were observed. Treatment with *A. fumigatus* improved plant response to the resistance against the invasion of pathogenic fungi by increasing the thickness of the cell wall and, thus, preventing pathogen penetration. This increase in thickness occurs as a result of the sedimentation of substances that are difficult to penetrate by fungi, such as lignin; thus, this thickening wall limits or progresses the pathogen or prevents its advancement [103].

## 4. Conclusions

The present study investigated the effect of endophytic *A. fumigatus* on the growth and aflatoxins produced by *A. flavus*. Endophytic *A. fumigatus* showed a noticeable ability to inhibit the growth and decrease aflatoxins production, particularly AFB_1_. Moreover, endophytic *A. fumigatus* improved the infected maize growth, as well as its redox status. At the level of the cell, TEM analysis exhibited that the plant could resist *A. flavus* by increasing the thickness of the cell wall and, thus, prevent fungal penetration, where a recovery in structural features was recorded. Eventually, the application of endophytic *A. fumigatus* treatment could be commercially used for controlling *A. flavus* growth and aflatoxins production, as well as their subsequent effects on maize growth.

## Figures and Tables

**Table 1:** In-vitro inhibition percentages of aflatoxins using endophytic *A. fumigatus.*

Aflatoxin Type	Aflatoxin (ng/mL)		Inhibition %
	Control	Treated	
**AFG_1_**	6.88	1.40	79.6
**AFG_2_**	ND	ND	ND
**AFB_1_**	3647.15	332.50	90.90
**AFB_2_**	99.39	6.80	93.10

ND means not detected.

**Table 2:** Inhibition percentages of aflatoxins using *A. fumigatus* in vivo.

Aflatoxin Type	Aflatoxin (ng/g)			Inhibition %
	Control Healthy	Control Infected	Treated with *A. fumigatus*	
**AFG_1_**	ND	ND	ND	ND
**AFG_2_**	ND	1.11	ND	100.00
**AFB_1_**	4.60	794.09	462.57	41.74
**AFB_2_**	ND	4.37	1.63	62.70

ND means not detected.

**Table 3:** Effect of *A. flavus* infection and *A. fumigatus* on germination % and shoot and root lengths in corn seeds.

Treatment	Germination %	Shoot Length (cm)	Root Length (cm)
**Healthy control**	87 ± 1.00 ^a^	13.66 ± 1.00 ^a^	5.66 ± 0.57 ^a^
**Infected control**	40.66 ± 1.52 ^c^	6.00 ± 1.00 ^b^	1.83 ± 0.28 ^b^
**Infected treated with** ***A. fumigatus***	76.66 ± 0.57 ^b^	13.0 ± 1.53 ^a^	6.33 ± 0.58 ^a^
**LSD at 0.05**	2.2	2.4	0.99

Data are expressed as means ± standard deviations of triplicate assays. The different alphabetic superscripts in the same column are significantly different (*p* < 0.05).

## Data Availability

The datasets generated and/or analyzed during the current study are available from the corresponding author on reasonable request.
